# Cohen Syndrome: Review of the Literature

**DOI:** 10.7759/cureus.3330

**Published:** 2018-09-18

**Authors:** Jonathan M Rodrigues, Hermina D Fernandes, Carrie Caruthers, Stephen R Braddock, Alan P Knutsen

**Affiliations:** 1 Internal Medicine, University of North Dakota School of Medicine and Health Sciences, Bismarck, USA; 2 Allergy and Immunology, The Vancouver Clinic, Vancouver, USA; 3 Pediatrics, Saint Louis University School of Medicine, Saint Louis, USA

**Keywords:** severe congenital neutropenia, cohen syndrome, vps13b, coh1, mishosseini-holmes-walton syndrome, hypotonia, myopia

## Abstract

Cohen syndrome was initially described as a syndrome including obesity, hypotonia, mental deficiency, and facial, oral, ocular and limb anomalies. Leukopenia, especially neutropenia, was later described as a feature of Cohen syndrome. Cohen syndrome is caused by an autosomal recessive (AR) mutation of the vacuolar protein sorting 13 homolog B (VPS13B, also referred to as COH1) gene on chromosome 8q22.2.

## Introduction and background

Cohen syndrome was first described by M. Michael Cohen Jr. in two affected siblings and one isolated case and later confirmed by Carey et al. in four patients with features similar to those described by Cohen, including obesity, hypotonia, mental deficiency, and craniofacial, ocular, and limb anomalies [1-2]. Norio et al. later identified neutropenia as a consistent feature of Cohen syndrome [3]. Since then, over 100 cases of Cohen syndrome have been described, with 35 cases from Finland, where the disease has shown greater homogeneity. Cohen syndrome tends to be more heterogeneous with a wide phenotypic variability outside the Finnish cohort. Although some patients with clinical features similar to Cohen syndrome were initially described to have “Mishosseini-Holmes-Walton syndrome”, it is now believed that these patients actually had Cohen syndrome, representing heterogeneity within the same syndrome [4-6].

## Review

Cohen syndrome (Online Mendelian Inheritance in Man (OMIM) entry number 216550) is caused by an autosomal recessive (AR) mutation of the vacuolar protein sorting 13 homolog B (VPS13B, also referred to as COH1) gene on chromosome 8q22.2. VPS13B is a transmembrane protein that is thought to function in vesicle-mediated transport and sorting of proteins within the cell and plays a role in the development and the function of the eye, hematological system, and central nervous system. Characteristic clinical features of Cohen syndrome are well-described and involve multiple systems as discussed below [7-10].

Perinatal

In the first report by Cohen et al., two of the three patients had decreased fetal activity [1]. This has been a consistent finding with as many as 50% of patients having decreased fetal activity [1-2, 7]. Most children are born at term, but the birth weight and length are often in the 10th to 25th percentile [9]. Hypotonia can be an apparent feature in infancy and can cause significant breathing and feeding difficulty [1, 7, 10-11]. Some authors have reported a high-pitched cry possibly secondary to laryngeal abnormalities, like that seen in 5p deletion (Cri-du-chat) syndrome [9, 12-14].

Growth

While low birth weight and short stature may be present, they are not essential features. Truncal obesity may develop in teenage years. It has been suggested that the term “obesity” be replaced with “abnormal truncal fat distribution” as these patients often present with an increased waist circumference but a normal body mass index (BMI) [15]. Functional studies have shown that the increased fat accumulation in patients with Cohen syndrome is due to an increased propensity of pre-adipocytes lacking VPS13B to differentiate into fat-storing cells [15]. An increased response of cells to insulin in earlier stages of differentiation leads to an accelerated expression of specific adipogenic genes [15].

Development 

There is a significant delay in attaining motor milestones with the ability to walk independently arising between two to five years of age [7]. In addition, there may be a language delay with the first words uttered between one to five years of age. Many cannot speak in full sentences by age of six years [7]. All patients with Cohen syndrome have some level of intellectual disability, with up to 22% having profound delay [7]. Disordered social interactions, including difficulty making friends, using non-verbal communication, understanding feelings of others, and sharing are common [16]. Patients may have difficulty with independence and self-help, although affected individuals are usually able to eat and use the toilet independently [12, 17]. These abnormalities seem to have an equal preponderance in males and females [16]. A cheerful disposition, friendly personality, and a high-pitched voice have also been reported [3, 7]. Antisocial, violent, destructive, rebellious, or untrustworthy behavior is rare [12]. Some children with Cohen syndrome may also be found to meet diagnostic criteria for an autism spectrum disorder [18-19]. Initiation of early intervention programs in physical therapy and occupational therapy to address motor delays and hypotonia, as well as speech/language therapy, is imperative [14]. Many cases can communicate successfully through sign language [12].

Craniofacial abnormalities

One of the inclusion criteria for the diagnosis of Cohen syndrome is the “typical facial characteristics,” including microcephaly, down-slanting palpebral fissures, wave-shaped palpebral fissures, hypertelorism, thick eyebrows, thick bushy hair, low hairline, long and thick eyelashes, very short philtrum, prominent upper central incisors, open mouth appearance due to a short upper lip, maxillary hypoplasia, micrognathia, high and narrow palate, prominent root of the nose, bulbous nasal tip, and thick and poorly folded earlobes or small or absent lobules of ears [3, 7, 10].

Dentition

In addition to the characteristic prominent upper incisors, patients may have an early periodontal breakdown, extensive alveolar bone loss, and often harbor putative pathogens more likely to be associated with periodontitis [7, 20]. A critical concern in patients with Cohen syndrome is the possibility of a difficult airway caused by characteristic craniofacial deformities and prominent upper incisors. During procedural anesthesia, it may be prudent to have the equipment to manage a difficult airway and an otolaryngologist available to provide a surgical airway if needed [21].

Ophthalmologic

There is progressive deterioration of vision throughout life, with early onset of progressive high-grade myopia, often requiring corrective lenses as early as two years of age. Progressive constriction of visual fields worsens visual function by the second decade of life [22-23]. Many affected adults have significant visual impairment by the age of 40 years, although total blindness is not common [7]. Myopia is mostly of the refractive-type due to high corneal and lenticular power, as a result of dysgenesis and atrophy of the cornea, ciliary body, and iris, causing iridial and zonular laxity and spherophakia [22]. Symptoms and findings in Cohen syndrome resemble retinitis pigmentosa [22, 24-26]. Other findings include mottled pigmentation of the retina, microphthalmia, microcornea, strabismus, astigmatism, shallow anterior chamber, sluggish pupillary reaction, retinal degeneration, bull’s eye maculopathy, optic atrophy, chorioretinal dystrophy, peripapillary atrophy, cortical lens opacities, lens subluxation, constricted visual fields, exophthalmos, keratoconus, nyctalopia, down-slanting palpebral fissures, ptosis, and coloboma [7, 22-26]. Although rare, acute angle closure glaucoma has also been reported [24]. Retinal dystrophic changes are progressive, and ultimately, vision may be limited to counting fingers and light perception [23]. Electroretinograms often show attenuated or extinguished responses [24-25]. Early correction of visual defects, such as glasses to correct refractive errors or strabismus, has a positive effect on development [24-26]. However, there is no available effective treatment to halt the progression of pigmentary retinopathy. Patients should have periodic and detailed ophthalmologic exams to evaluate for refractive errors or retinal dystrophy.

Hematologic

Leukopenia, especially neutropenia, is a common feature of Cohen syndrome. Severe congenital neutropenia (SCN) is often present from birth and is mild to moderate, noncyclic, and non-fatal [7, 27]. Patients may respond to bacterial infections with neutrophilic leukocytosis [27]. Although SCN may be without severe bacterial infections, other patients may have recurrent infections, aphthous ulcers, and chronic or recurrent gingivitis [27]. Bone marrow cellularity is usually normal or increased. De Ravel et al. described one patient with presumed Cohen syndrome and asymptomatic persistent thrombocytopenia; however, this finding has not been described elsewhere in the literature [28]. Hypercoagulability with deficient protein C, protein S, and anti-thrombin III complicated by severe thrombosis have been described in two siblings; however, the diagnosis of Cohen syndrome was not firmly established by molecular testing in this report [29]. Neutropenia is correctible using recombinant human granulocyte colony stimulating factor (rHG-CSF) [14, 27]. Use of rHG-CSF would be warranted in patients with SCN and recurrent infections and/or recurrent aphthous ulcers. These individuals also need serial absolute neutrophil count (ANC) determinations to monitor for neutropenia [14].

Gastrointestinal

Feeding difficulties as neonates have been reported in as high as 75% of patients [7].

Musculoskeletal

Most patients with Cohen syndrome have slender hands and feet. Hypotonia is often first noticed in the neonatal period but becomes obvious by one year of age. Spasticity may develop at a later stage [7]. Various other musculoskeletal deformities may be seen, including cubitus valgus, genu valgum, pes planovalgus, kyphosis, scoliosis, ligamentous laxity, and articular hypermobility, many being secondary to underlying muscular hypotonia. These patients may also have single transverse palmar creases, thenar and hypothenar hypoplasia, mild syndactyly, a wide gap between first and second toes, and lumbar lordosis [10]. Juvenile rheumatoid arthritis has also been reported in association with Cohen syndrome [28].

Neurologic

Relatively consistent features among patients include motor incoordination or “clumsiness”, brisk tendon reflexes, and muscular hypotonia [3, 7, 10]. Cerebellar hypoplasia has also been reported [30]. A magnetic resonance imaging (MRI) study done to rule out other causes of mental retardation may show an enlarged corpus callosum, which supports the diagnosis [7, 31]. Seizures and electroencephalographic (EEG) abnormalities are not typical features of Cohen syndrome, although these have been described in some cases [1, 11]. Patients may have low-voltage, non-irritative EEGs [7].

Cardiac

Cardiac defects reported in Cohen syndrome include decreased left ventricular function with advancing age, valvular defects (such as a floppy mitral valve and mitral regurgitation), vascular defects including a dilated descending aorta, cardiac systolic murmurs, ST segment abnormalities (ST-segment depression, T-wave inversion), essential hypertension, and pulmonary hypertension [3, 7, 29, 32-34]. Patients also tend to have decreased high-density lipoprotein (HDL) levels and often meet several criteria for metabolic syndrome [15].

Endocrine

Delayed onset of puberty is typical [2]. North et al. described identical twin girls with Cohen syndrome with precocious puberty, although this is not typical [35]. Gonadotropin deficiency, growth hormone deficiency, insulin resistance, non-insulin-dependent diabetes mellitus, and cryptorchidism have been described [1, 11, 35-40]. After elevated fat accumulation in VPS13B-deficient cells, insulin resistance is observed through a reduction in phosphorylation of AKT (a protein kinase), which may explain the impaired glucose tolerance in some Cohen syndrome patients [15]. Thus, it may be important to monitor blood pressure, lipid metabolism parameters, fasting blood glucose levels, and glycated hemoglobin (A1C) annually. Furthermore, older patients may have an abnormal glucose tolerance despite relatively normal fasting blood glucose levels. It may, therefore, be prudent to perform oral glucose tolerance tests in adolescence, and every five years thereon.

Genetics

Cohen syndrome is an autosomal recessive disorder first mapped to the Chediak-Higashi syndrome gene (CHS1) locus of chromosome 8 by Tahvanainen et al. in 1994 [41]. COH1 (an ortholog of the VPS13B protein in Saccharomyces cerevisiae) is transcribed from 62 exons spanning a genomic region of 864 kb and encodes a transmembrane protein the vacuolar protein sorting 13 homolog B (VPS13B, COH1) gene on chromosome 8q22.2 [8, 42]. The translated protein VPS13B has a molecular weight of 44.8 kilodaltons (kDa) comprised of 4,022 amino acids. VPS13B is a transmembrane protein that is thought to function in vesicle-mediated transport and sorting of proteins within the cell and plays a role in the development and the function of the eye, hematological system, and central nervous system. By forming a physical and functional complex with the small GTPase RAB6 at the Golgi complex, VPS13B co-localizes with the cis-Golgi matrix protein GM130 and is integral for maintaining the structural and functional integrity of the Golgi complex [43-44]. VPS13B was also shown to play a crucial role in Golgi protein glycosylation and in endosomal-lysosomal trafficking [45]. The mechanism by which abnormalities in this protein lead to the phenotype of Cohen syndrome is currently unknown.

Cohen syndrome is a rare syndrome worldwide, but a higher concentration has been described in the Finnish, Japanese, Caucasian, Ohio Amish, Lebanese, and Jewish populations. In the latter group, the diagnosis has been controversial. A “Baloch” variant has been described in three large consanguineous Pakistani families [46]. Broad phenotypic variability has made the diagnosis of Cohen syndrome challenging. There is no generalized consensus on diagnostic criteria for Cohen syndrome. Horn et al. proposed the presence of at least three major criteria (intellectual disability, short stature, hypotonia, microcephaly, chorioretinal dystrophy, and narrow hands and feet) and one minor criterion (truncal obesity, neutropenia, myopia, or facial abnormalities) to establish the diagnosis of Cohen syndrome [13]. Clinical recognition of the distinctive facial dysmorphism is difficult prior to the age of six years [47-49]. Thus, it is difficult to diagnose Cohen syndrome in younger children. To overcome this obstacle, Chandler et al. proposed that in addition to significant learning disabilities, a child with Cohen syndrome had to have at least two of the following features: facial gestalt, pigmentary retinopathy, or neutropenia (< 2 x 10^-9^/mm^3^) [48]. Kolehmainen et al. proposed that patients having six of eight clinical criteria (developmental delay, microcephaly, typical Cohen syndrome facial gestalt, truncal obesity with slender extremities, overly sociable behavior, joint hypermobility, high myopia and/or retinal dystrophy, and neutropenia) could be considered to have true Cohen syndrome [8]. Patients fulfilling five or fewer criteria were considered to have “Cohen-like syndrome”.

While 22 different VPS13B pathogenic genetic variants were identified in patients having “Cohen syndrome”, no VPS13B pathogenic variants were identified in patients who only had “Cohen-like syndrome”. Falk et al. showed that “facial gestalt” alone is an unreliable indicator of Cohen syndrome as there can be significant variability between ethnic populations [9, 14]. In contrast, features (such as retinal dystrophy, progressive high-grade myopia, microcephaly, hypotonia, joint hypermobility, intellectual disability, and global developmental delay) are consistent in patients with Cohen syndrome across ethnicities and are strong clinical indicators for establishing a diagnosis [9, 14]. In a study by El Chehadeh et al., all patients with VPS13B mutations had either chorioretinal dystrophy or neutropenia [50]. This study also estimated that the Kolehmainen criteria had 100% sensitivity and 77% specificity in identifying Cohen syndrome. 

## Conclusions

In summary, it is important to consider a diagnosis of Cohen syndrome in children with microcephaly who present with early-onset hypotonia, neutropenia, and global developmental delay. Ophthalmologists should consider the diagnosis of Cohen syndrome in a young child with developmental delay, severe myopia, nyctalopia, and pigmentary retinopathy. The distinctive facial dysmorphism is a leading clue to establishing a diagnosis. In such children, a detailed ophthalmological exam, a complete blood count, and brain MRI should be considered. Physicians should also be mindful of complications arising from neutropenia, poor dentition, difficult airway encountered during anesthesia, and feeding difficulties as these may warrant special attention and care. 
